# Kinematic analysis of an unrestrained passenger in an autonomous vehicle during emergency braking

**DOI:** 10.3389/fbioe.2024.1270181

**Published:** 2024-03-12

**Authors:** Silvia Santos-Cuadros, Álvaro Page del Pozo, Carolina Álvarez-Caldas, José Luis San Román García

**Affiliations:** ^1^ Mechanical Engineering Department, Instituto de Seguridad de los Vehículos Automóviles (ISVA, Institute for Vehicle Safety Assurance), Carlos III University of Madrid, Leganés, Spain; ^2^ Instituto Universitario de Ingeniería Mecánica y Biomecánica, University Institute of Mechanical and Biomechanical Engineering, Universitat Politècnica de València, Leganés, Spain

**Keywords:** Biomechanics, human movement analysis, joint kinematics, cervical injury, road safety, autonomous braking, unrestrained passenger

## Abstract

Analyzing human body movement is a critical aspect of biomechanical studies in road safety. While most studies have traditionally focused on assessing the head-neck system due to the restraint provided by seat belts, it is essential to examine the entire pelvis-thorax-head kinematic chain when these body regions are free to move. The absence of restraint systems is prevalent in public transport and is also being considered for future integration into autonomous vehicles operating at low speeds. This article presents an experimental study examining the movement of the pelvis, thorax and head of 18 passengers seated without seat belts during emergency braking in an autonomous bus. The movement was recorded using a video analysis system capturing 100 frames per second. Reflective markers were placed on the knee, pelvis, lumbar region, thorax, neck and head, enabling precise measurement of the movement of each body segment and the joints of the lumbar and cervical spine. Various kinematic variables, including angles, displacements, angular velocities and accelerations, were measured. The results delineate distinct phases of body movement during braking and elucidate the coordination and sequentiality of pelvis, thorax and head rotation. Additionally, the study reveals correlations between pelvic rotation, lumbar flexion, and vertical trunk movement, shedding light on their potential impact on neck compression. Notably, it is observed that the elevation of the C7 vertebra is more closely linked to pelvic tilt than lumbar flexion. Furthermore, the study identifies that the maximum angular acceleration of the head and the maximum tangential force occur during the trunk’s rebound against the seatback once the vehicle comes to a complete stop. However, these forces are found to be insufficient to cause neck injury. While this study serves as a preliminary investigation, its findings underscore the need to incorporate complete trunk kinematics, particularly of the pelvis, into braking and impact studies, rather than solely focusing on the head-neck system, as is common in most research endeavors.

## 1 Introduction

In addition to the indisputable human toll of lives lost in road accidents (approximately 1.3 million people), there are significant economic repercussions for society. These costs can amount to as much as 3% of a country’s Gross Domestic Product if we consider the number of non-fatal victims (around 50 million people). Many of these non-fatal injuries can result in long-term disabilities or lasting effects, severely impacting quality of life for the victims. Indeed, road crashes are recognized by the World Health Organization (WHO) as a major global public health concern, which is often described as a silent epidemic. According to the WHO, nearly 1.3 million road deaths and 500 million injuries are preventable (Bansode and Tanriover, 2018). Advanced Driver Assistance Systems (ADAS) play a pivotal role in addressing this issue. The European Union anticipates that 25,000 fatalities and approximately 140,000 serious injuries could be averted by 2038 through the mandated implementation of certain ADAS by vehicle manufacturers (Union PO of the E, 2018). Notably, the Autonomous Emergency Braking (AEB) system, required in all new vehicle type-approvals in the European Union since July 2022, is highlighted among these systems. Consequently, the proliferation of vehicles equipped with this capability is expected to rise. Given that these systems can prevent collisions, through autonomous emergency braking and thereby influence passenger kinematics (such as altering posture before an event) (Jakobsson et al., 2006; Bose et al., 2010; McMurry et al., 2018), incidents where collisions are avoided altogether are likely to become more common. However, to date, it is noteworthy that the majority of studies on body movement during road traffic accidents have primarily focused on the impact phase, where the injury mechanism may differ significantly. Moreover, it is important to recognize that the AEB system could have a substantial impact on all road traffic injuries, particularly considering that whiplash-associated disorders constitute 60% of permanently disabling injuries among vehicle occupants (Malm et al., 2008), with the majority occurring in low-speed incidents (Kullgren and Krafft, 2007). Therefore, given the prospective scenario where impacts are averted, especially at low speeds, there is a pressing need to closely examine what transpires kinematically during the pre-impact phase, which coincides with the braking stage.

To develop safety systems, Anthropomorphic Test Devices (ATDs) and Post Mortem Human Surrogates (PMHSs) are typically utilized (Beeman et al., 2012; Yoganandan et al., 2017; Albert et al., 2018; Meyer et al., 2019). This approach is logical when examining moderate to severe impact scenarios, where it is assumed that muscle response has minimal influence on passenger kinematics. Conversely, when assessing the effectiveness of integral safety systems like emergency braking, muscle function plays a crucial role in passenger movement during low acceleration situations (Ejima et al., 2007; Ejima et al., 2008; Ejima et al., 2012). However, ATDs and PMHSs have limited biofidelity, particularly regarding muscle response. Thus, it is imperative that models simulating passenger kinematics in pre-crash scenarios undergo validation based on studies involving volunteers subjected to mild loading conditions, which necessitate high muscle biofidelity (Beeman et al., 2012; Kirschbichler et al., 2014). Given the significant influence of muscles on overall kinematic response at low speeds (Santos cuadros et al., 2021), this study will involve 18 volunteers to achieve the highest possible muscle biofidelity. Consequently, the results of this research could be instrumental in evaluating and validating human kinematics simulation models. Moreover, the type of restraint system significantly impacts passenger response during braking, as its primary function is to prevent excessive occupant motion. The majority of studies (McConnell et al., 1995; Szabo and Welcher, 1996; Watanabe et al., 2000; Kumar et al., 2003a; Kumar et al., 2005a; Kumar et al., 2006; Beeman et al., 2011; Carlsson and Davidsson, 2011; Beeman et al., 2012; Ito et al., 2013; Ólafsdóttir et al., 2013; Östh et al., 2013; Kirschbichler et al., 2014; Reed et al., 2018; Graci et al., 2019; Holt et al., 2020) in this field involve volunteers restrained by a three-point seat belt or similar systems, which limit torso and pelvis movement during deceleration. To date, there is a lack of research evaluating kinematics during emergency braking with unrestrained seated volunteers. While studies with unrestrained passengers have been conducted using ATDs, PMHSs or computational models, the emergence of autonomous vehicles considering seats without the need for seat belts at low speeds raises pertinent questions. This absence of restraint systems is also typical in public transport scenarios (buses, metro, etc.), where the risk of death or serious injury is seven to nine times lower for bus and coach passengers compared to other vehicle users (Albertsson and Falkmer, 2005). Bus and coach fatalities account for 0.3%–0.5% of all traffic deaths in Europe. Nevertheless, it should be noted that among the leading causes of injury on public transport is emergency braking, along with boarding (Albertsson and Falkmer, 2005; Björnstig et al., 2005; Palacio et al., 2009; Henezi and Winkler, 2023). Additionally, while design and improvements of public transport are more focused on service or comfort (Eboli et al., 2016; Barabino et al., 2019) in such a way that more research is being done on how sharp decelerations are perceived in terms of the passenger’s sense of comfort, the biomechanical analysis of injury mechanisms involved in these abrupt speed changes that are so typical in urban buses is lacking. Moreover, while most studies about public transport tend to focus on collisions and rollovers (Olivares and Yadav, 2009; Olivares, 2015), the biomechanical analysis of injury mechanisms in non-collision scenarios is not well understood (Palacio et al., 2009; Lutin et al., 2016). Therefore, there is a need to analyze the kinematics when passengers lack restraint systems during emergency braking, particularly regarding the influence of the pelvis on overall injury risk at low speeds, where no lap belt supports the pelvic area. Hence, in this study, the subjects do not use any seat belts.

Although all parts of the body interact to produce a global body response during emergency braking, the existing studies on body movement during a road traffic accident (Panjabi et al., 2004; Ejima et al., 2007; Behr et al., 2010; Carlsson and Davidsson, 2011; Ejima et al., 2012; Rooij et al., 2013; Ólafsdóttir et al., 2013; Östh et al., 2013; Kuo et al., 2018; Holt et al., 2020; Ghaffari and Davidsson, 2021; Larsson et al., 2022) have predominantly focused on evaluating the head-neck system or the kinematics of the head and torso separately, neglecting the role of the pelvis, which is typically supported by the seat belt. In addition, they often present results independently without analyzing possible coordination between the movement of the different anatomical regions. The head and neck are usually analyzed separately, without considering the action that may be caused by the torso (generally restrained by the belt) and the pelvis, whose movement is almost completely ignored. To enhance current road safety systems it is crucial to analyze the complete kinematic chain, study coordination between movements and understand the relationship between forces acting on the neck and the movement of each body segment. Therefore, this study aims to evaluate the entire body’s response, analyzing the movement of the pelvis-thorax-head chain comprehensively. The study of the thorax and pelvis movement is especially relevant to understanding the acceleration that occurs in the head due to the collision of the trunk against the seatback when it travels backward again after the maximum excursion of the body forward due to braking. The existing studies typically focus on the entirety of the forward excursion, constrained by the seat belt; however, in its absence, the peak head acceleration occurs post-braking, with the vehicle fully stopped, when the trunk rotates backward and impacts the seat back. Another objective of this research is to scrutinize the magnitude of this acceleration and its potential implications for neck injury. Analyzing trunk and head kinematics in braking scenarios without seat belts could inform the development and validation of human models for vehicle occupants, particularly those aiming to account for pre-crash responses. These data will enhance our understanding of passenger reactions, injury risks and the efficacy of restraint and road safety systems (such as AEB) in future autonomous mobility scenarios, particularly during pre-impact scenarios, evasive maneuvers like emergency braking and low-speed impacts.

This study presents an experimental investigation wherein the movement of the head, neck, torso and pelvis in seated, unrestrained passengers is collectively analyzed during emergency braking in an autonomous vehicle. To achieve this, a sample of 18 volunteers was examined. The movement was captured using a video analysis system operating at 100 frames per second, using reflective markers positioned on the aforementioned anatomical regions. This setup enabled precise measurement of each body segment’s movement and facilitated joint kinematics analysis.

This study proposes two hypotheses. Firstly, it assumes that head movement during emergency braking (without a restraint system) is coordinated with thorax and pelvis movements, which contribute to trunk elevation during forward movement and may impact injury risk. Secondly, it suggests that the rebound phase after maximum body excursion due to braking entails greater head accelerations, potentially indicating a higher risk of cervical injury. Therefore, it is essential to analyze the rebound phase alongside maximum body flexion in emergency braking or low-speed impacts, when passengers are unrestrained.

## 2 Materials and methods

This section describes the study sample, the experimental setup, the equipment used, and how the data were analyzed.

### 2.1 Description of the study sample

Eighteen healthy volunteers (8 women and 10 men) participated in this study. The inclusion criterion for participation in the trials was the absence of any pre-existing injuries that could be exacerbated or cause additional injuries during the experiment, particularly cervical injuries. The age of the volunteers ranged from 22 to 54 years, with an average of 31.9 ± 8.8 years. Their weights ranged from 47 to 90 kg, with an average of 66.3 ± 13.1 kg; and heights ranged from 154 to 189 cm, with an average of 170.8 ± 9.9 cm.

### 2.2 Experimental setup

To create a realistic and bio-faithful environment, an autonomous bus without a driver’s seat (EasySmile EZ10 model) was utilized. Volunteers were seated facing the direction of travel within the bus, without any restraint system. Autonomous emergency braking was triggered at a predetermined moment (unknown to the passenger).

The trial was meticulously designed to ensure the safety and wellbeing of all participants, and steps were taken to monitor their health status throughout. It should be noted that the experiment was conducted at low speeds to maintain safety levels below the injury threshold. However, in the event that a participant experienced any pain or discomfort during the tests, they were instructed to promptly inform the team to halt the experiment.

**FIGURE 1:**
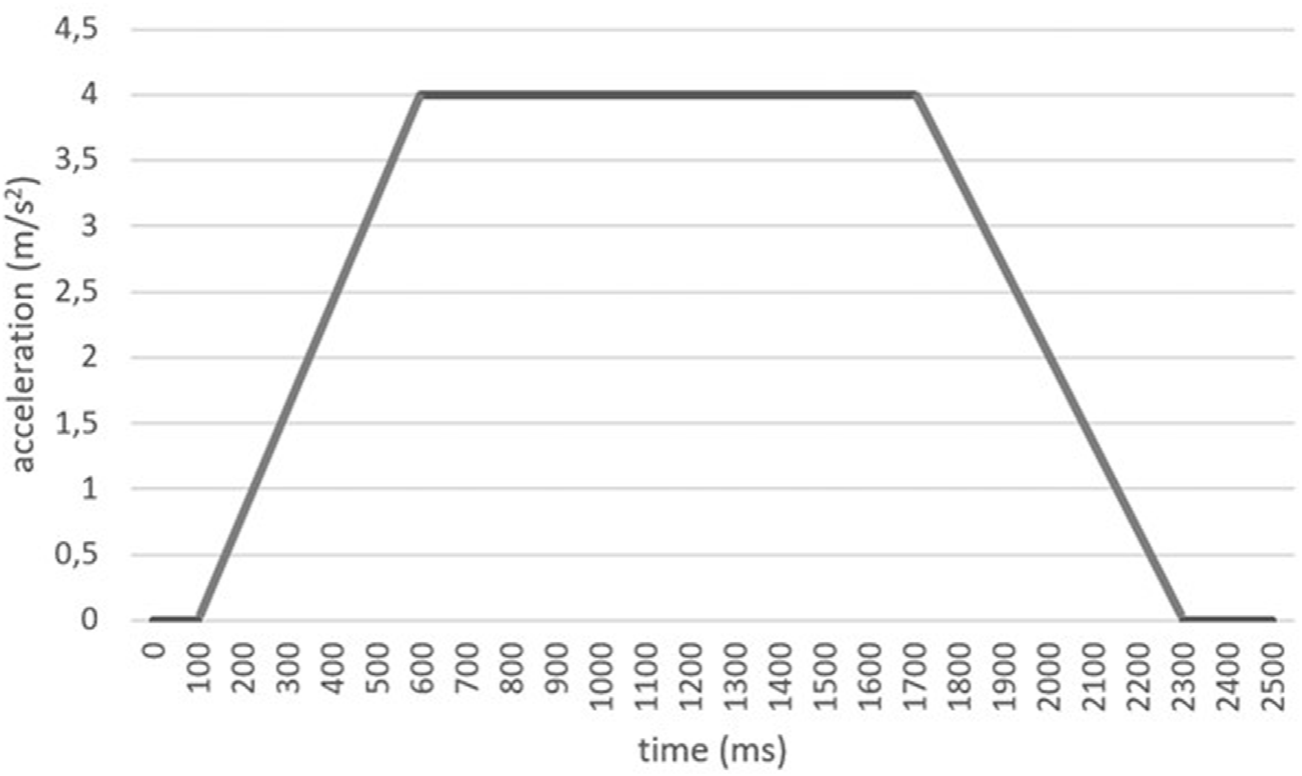
Deceleration curve programmed in the autonomous vehicle during emergency braking.

The detailed steps followed in the development of this study are outlined below:

• Step 1: Trial explanation and Informed consent). The research team explained to the volunteer (both in writing and orally) what the test consisted of and the associated risks. Upon agreeing to participate, volunteers were required to sign an informed consent form. Notwithstanding this, participants retained the freedom to withdraw from the study at any point during the experiment. The protocol followed in this research meets the requirements for research involving human subjects according to the Declaration of Helsinki. Approval was obtained from the Ethics Committee of the Carlos III University of Madrid where the trials were conducted.
• Step 2: Pre-test questionnaire. Before the trial commenced, the volunteers completed a questionnaire designed to assess their health status and ascertain the absence of any injuries that might elevate the risk of injury during the experiment. Additionally, anthropometric data such as height, weight, gender, and age were recorded at this stage.
• Step 3: Volunteer instrumentation. The volunteers were equipped with reflective position markers and surface electromyography (sEMG) sensors. These markers were placed on various body parts including the head, neck (C7), shoulder, thorax, lumbar region, pelvis and knee, enabling monitoring of the subject’s movement in their sagittal plane during emergency braking. The movement of these markers was recorded by a high-speed camera (SONY DSC-RX0) at 100 frames per second. A video analysis system made it possible to measure the movement of each body segment and to analyze the joint kinematics. Furthermore, sEMG sensors were positioned to capture muscle responses from superficial neck muscles: the trapezius and sternocleidomastoid muscles. This was achieved through a palpation test to locate the middle and the upper parts of the muscle belly. The data collected from these sensors were utilized to ensure that volunteerskept their cervical muscles relaxed during vehicle operation prior to braking. The subjects were supposed to be relaxed before braking since they were unaware of the instant of braking. Signal normalization of the sEMG signals was conducted based on mean activation levels obtained during the task, as explained in (Pons et al., 2013; Del Toro et al., 2020).
• Step 4: Initial posture standardization. To ensure uniformity in their starting positions, the volunteers were instructed to align their gaze forward, ensuring alignment of the head and torso. They were also directed to maintain contact between their backs and the seatback, with arms resting on their legs forming approximately a 90-degree angle between the arm and forearm. The research personnel supervised adherence to this starting position prior to initiation of the test. Once the subject was seated, the volunteers were asked to indicate the position of their iliac crest and ischial tuberosity to accurately position and align two pelvic support markers.
• Step 5: Autonomous emergency braking test and Data acquisition. The emergency braking tests were designed following several references (Siegmund et al., 2003; Kumar et al., 2005b; Olive et al., 2019; Holt et al., 2020). In no case did the subject use any seat restraint system. Initially, the volunteers assumed a neutral seated position, facing forward with no initial relative head-neck angle, while engaging in conversation with a person in front of them. Emergency braking was initiated by the autonomous bus while the volunteer remained seated in the forward-facing direction, without a seatbelt. The volunteers were instructed to keep their muscles relaxed, a condition reiterated before the start of the test. The precise moment of braking was unknown to the volunteer. The test concluded once the subject (after maximum forward body excursion and subsequent rebound to the rear) remained stationary in the seat. The vehicle was programmed to achieve a deceleration of 4 m/s^2^. This value was chosen after consulting prior studies on braking tests with subjects (McConnell et al., 1993; McConnell et al., 1995; Ono and Kanno, 1996; Kumar et al., 2002; Kumar et al., 2003b; Kumar et al., 2005a; Hernández et al., 2005; Kumar et al., 2006; Carlsson and Davidsson, 2011; Kirschbichler et al., 2014), where the magnitudes vary between 4 and 12 m/s^2^. However, in all these previous research studies the subjects used restraint systems. Since our volunteers were unrestrained, the authors decided to include the minimum value used in the aforementioned works (4 m/s^2^) to prevent possible injuries during testing. The selected deceleration value aligns with emergency braking scenarios simulated in bus studies (De Graaf and Van Weperen, 1997; Palacio et al., 2009; Kirchner et al., 2014; Schubert et al., 2017; Soule et al., 2020; Vychytil and Špirk, 2020; Krašna et al., 2021; Keller and Krašna, 2023), where decelerations typically range from 1 to 4 m/s^2^. The deceleration profile of the autonomous vehicle during emergency braking was confirmed using an accelerometer (MAHA VZM 300) installed on the bus, as illustrated in Figure 1.
• Step 6: Post-test questionnaire. Following completion of the test, the volunteers were required to respond to a second questionnaire designed to assess whether they experienced any injuries or discomfort during the tests. This questionnaire was administered immediately after the test and repeated 24 h later to confirm the absence of any lingering discomfort.
• Step 7: Signal processing and Data analysis. All data collected during the tests underwent comprehensive analysis, which is detailed in the following section.

### 2.3 Data processing

Three rigid segments (pelvis, thorax and head) and two joints (lumbar and neck) were considered for the kinematic analysis. The movement of the segments was recorded by a set of technical markers fixed on each segment, whose coordinates were measured by the video analysis system. The coordinates of these markers are smoothed and derived numerically using the local regression algorithm described in (Page et al., 2006). From the smoothed coordinates, the linear and angular displacements of each segment and the corresponding velocities were calculated using the algorithms described in (Page et al., 2009a). These displacements and velocities refer to the absolute movement of the segments, taking as a reference the initial position at the beginning of braking.

**FIGURE 2:**
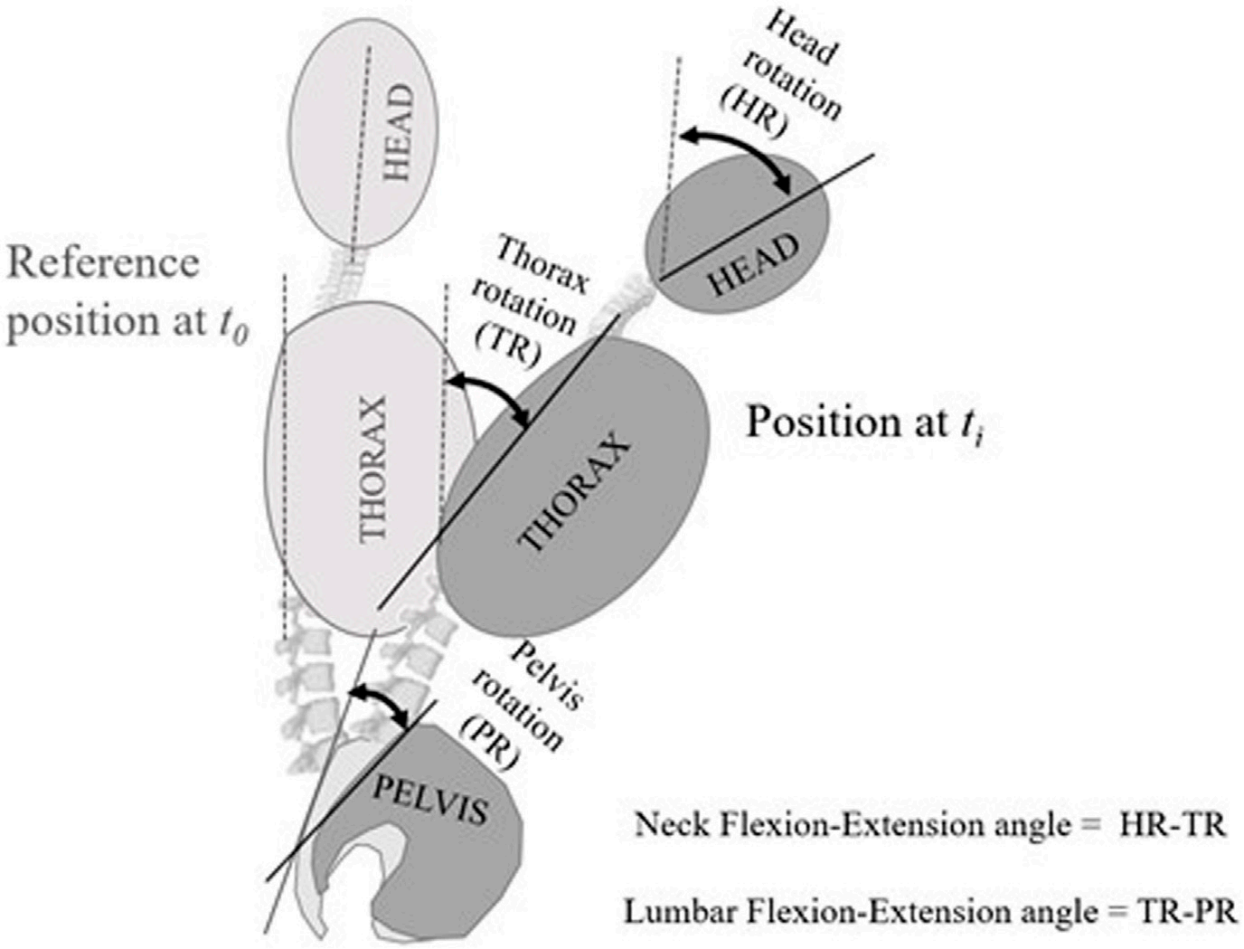
Measured angles. The rotation angles of each segment (pelvis, thorax and head) are measured from the angular displacement of the technical markers from the reference position at time t_0_ to the position at time t_i_. Clockwise turns (flexion) are negative; counterclockwise turns (extension) are positive. The flexion-extension angle of the neck is obtained by subtracting the rotation of the thorax from that of the head. The lumbar flexion-extension angle is obtained by subtracting the rotation of the pelvis from that of the thorax. Angles relative to anatomical landmarks are not taken; the neck or lumbar joint angles are measured from the initial reference position.

The neck and lumbar spine movements were calculated from the relative movement between the distal and proximal segments. Since the movement is planar, the neck’s rotation angle was calculated by subtracting the rotation of the thorax from that of the head (Venegas et al., 2020). Similarly, the lumbar angle was estimated as the subtraction of the absolute rotation of the thorax minus that of the pelvis (Page et al., 2009b) (see Figure 2).

Table 1 lists the calculated variables, divided into functional (functions of time) and numerical (ranges minimum and maximum values of the functional variables) variables. In addition to the variables associated with the movement of the segments and joints, the length of the neck, the displacement of the marker located on C7 and the displacement of the auricular marker with respect to C7 were also calculated. The maximums, minimums and range of each numerical variable were calculated for each subject from the individual movement curves. Angular displacements and velocities are positive for extension movement and negative for flexion movement.

**TABLE 1:** Kinematic, functional and numerical variables that appear in the analysis.

Magnitude	Functional variables	Numerical variables
**Angles** (°)	Head rotation	Maximum head flexion and extension
	Thorax rotation	Head flexo-extension range
	Pelvis rotation	Maximum thorax flexion and extension
	Neck flexo-extension angle	Thorax flexo-extension range
	Lumbar flexo-extension angle	Maximum pelvis flexion and extension
		Pelvis rotation range
**Displacements**	Displacement of C7	Range
	Vertical displacement of the auricular marker with respect to C7	
**Neck length**	Vertical distance between C7 and the auricular (ear) canal	
**Angular velocities** (°/s)	Head angular velocity	Maximum head flexion velocity
	Thorax angular velocity	Maximum head extension velocity
	Pelvis angular velocity	Head flexo-extension velocity range
	Neck flexo-extension angular velocity	Maximum thorax flexion velocity
	Lumbar flexo-extension angular velocity	Maximum thorax extension velocity
		Thorax flexo-extension velocity range
		Maximum pelvis flexion velocity
		Maximum pelvis extension velocity
		Head flexo-extension velocity range

Since the durations of the movement phases were different in each subject, it was necessary to normalize the time scale to analyze the functional variables (Ramsay, 1997). A linear normalization (Duhamel et al., 2004) was used, changing the time in seconds for the normalized time (t_n_), which represents the percentage of time elapsed from the start of the movement to the reference point taken as t_n_ = 100. The instant at which the braking of the vehicle began was taken as the start of the movement and, the reference point (t_n_ = 100) was taken as the instant at which the maximum extension of the head occurred with respect to the initial position. This last instant was chosen because it appears in all the subjects, occurs at the end of the movement and is easily identifiable. This reference point is usually located after the thorax impacts against the seat back once the vehicle has stopped. In some subjects, there was more than one rebound, so there were several peak extension movements. In those cases, the first shock was used, which was usually the strongest. Logically, the movement of the head and trunk continues after this point, with a head flexion. This implies that there are records with values greater than 100. However, the analysis was limited to the values of t_n_ ∈ [0, 110], since 110 is the minimum value that t_n_ takes in all the records at the end of the movement. In any case, the 0–110 interval allows us to register the maximum neck extension and subsequent flexion, which in some cases ends with the head at rest and then continues with another extension-flexion-rest cycle.

The following milestones were identified on the standardized records and used to describe the movement and, consequently, kinematic curves:

• T0 = 0: Start of the measurement. This corresponds to the instant at which vehicle braking begins.
• T1: Instant of maximum angular velocity of thorax flexion, after braking has started and before reaching maximum flexion.
• T2: Instant of maximum thorax flexion.
• T3: Instant of maximum angular velocity of the thorax extension, before T4.
• T4 = 100: Instant of maximum head extension, after the thorax collides with the seat back.

In addition to the kinematic variables outlined in Table 1, we also estimated the forces exerted on the head. Utilizing the inverse dynamic model described in (Toro, 2022), we computed the head’s mass. This model takes into account the acceleration of the auricular marker positioned on the subjects’ heads, which approximates the head’s center of mass. The governing equation for this model is as follows:

$$Head\;mass\;\left(kg\right)=0.0137*Height\;\left(cm\right)+0.0504*Weight\;\left(kg\right)-0.2896$$

Lastly, the risk of cervical injury was assessed utilizing the Neck Injury Criterion (NIC) formulated by Boström et al. (Svensson et al., 1996). This criterion is founded upon the relative acceleration and velocity between the T1 and C1 vertebrae, as depicted in the following expression:

$$NIC=acceleration*0.2+{velocity}^{2}$$

The threshold for assuming cervical injury is set at 15 m^2^/s^2^. To evaluate this, data from both the C7 and auricular markers were employed.

### 2.4 Statistical analysis

The statistical analysis consisted of three stages. Firstly, a descriptive analysis of the numerical and functional variables was carried out. The numerical variables were described by their means and standard deviations. For each functional variable, a mean function was computed as the mean of the corresponding curves, point by point, across all volunteers (Ramsay, 1997; Duhamel et al., 2004).

$$\bar{x}\left(t_{ni}\right)=\frac{1}{N}\sum_{j}^{N}x_{j}\left(t_{ni}\right)\;\left(i=1,2,\ldots 110\right)$$

This mean represents the approximate shape of the curves, although it presents a certain cancellation effect due to the lags between curves. For this reason, the maxima of the mean function are somewhat lower than the mean of the maxima of the individual curves (Ramsay, 1997).

Secondly, the coordination between the movements of each segment was analyzed. To this end, Spearman’s correlation coefficients were calculated between the ranges of motion of three motions: the pelvis relative to the seat, the lumbar joint (thorax movement relative to the pelvis) and the neck joint (head movement relative to the torso). The coefficients were calculated for both rotation ranges and velocity ranges.

Finally, the contribution of the thorax and pelvic motion to the elevation of the trunk was contrasted. A regression model was adjusted for each subject using the instantaneous measurements between T0 and T2. The dependent variable was the height of C7, while the independent variables were the pelvis and thorax flexion-extension angles. In this way, it is possible to analyze whether each segment’s rotations contribute to the elevation of C7. For each adjustment, coefficients were obtained for each independent variable and the correlation coefficient, R, of the fitted model. The median and the interquartile range represent these coefficients. A Friedman test was performed to check whether the contributions of pelvic and thorax rotation were different.

## 3 Results

**FIGURE 3:**
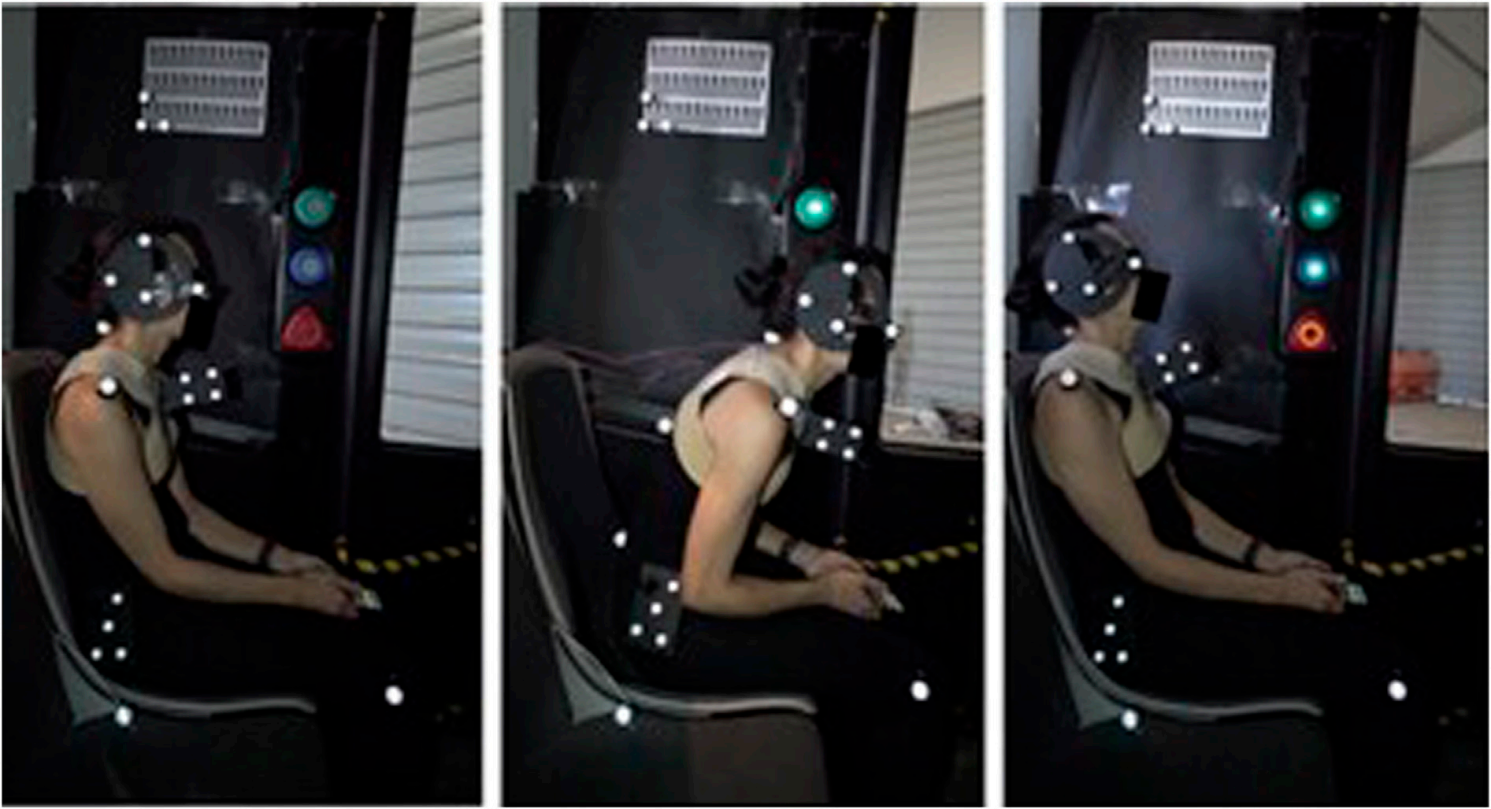
Movement of a subject during the emergency braking test (from left to right: starting position (T0), the instant of maximum thorax flexion (T2), the instant of maximum head extension (T4)).

Figure 3 shows an example of the movement of an unrestrained passenger during the emergency braking test.

Table 2 presents the characteristics of the participants involved in the study. Initially, 18 subjects underwent testing; however, one participant had to be excluded due to errors in the measurement system during the test. Consequently, the analysis was conducted on a final sample comprising ten men and seven women.

**TABLE 2:** Characteristics of the participants in the study.

Feature	Male (*n* = 10)	Female (*n* = 7)	Total (*n* = 17)
**Age** (years)	29.2 (10.19)	35.4 (6.9)	31.7 (9.3)
**Height** (cm)	177.5 (6.3)	161.9 (7.9)	171.1 (10.4)
**Weight** (kg)	70.1 (12.2)	61.2 (15.4)	66.4 (13.8)
**Length neck C7-Tragus** (cm)	10.8 (1.5)	11.1 (1.8)	10.9 (1.6)
**Length pelvis-C7** (mm)	52.5 (1.5)	46.6 (5.8)	50.1 (4.8)

Table 3 shows the time milestones described in absolute values and the normalized time scale. When braking begins, the thorax rotates forward until it reaches its maximum flexion velocity after 0.46 s, on average (20% of the normalized time). It then continues to flex until it reaches full flexion at T2 = 1.66 s (on average, t_n_ = 62.9% of the normalized time). Then, the trunk travels backward again rapidly, reaching the maximum angular velocity at T3 = 2.27 s (average, t_n_ = 86.5%). This is when the back collides with the seat back, and the extension movement slows down. However, the movement of the head accelerates, reaching its maximum extension at the instant T4 = 2.65 s (average, corresponding to t_n_ = 100%, as this is the reference point).

**TABLE 3:** Time instants of the events taken as reference points.

Event	Time (s) from T_0_. Mean (std)	Normalized time t_n_ (%) from T_0._ mean (std)
**T**_**1**_ (Maximum angular velocity of thorax flexion)	0.46 (0.23)	20.4 (6.8)
**T**_**2**_ (Maximum thorax flexion)	1.66 (0.33)	62.9 (11.8)
**T**_**3**_ (Maximum angular velocity of the thorax before tn = 100)	2.27 (0.21)	86.5 (6.0)
**T**_**4**_ (Maximum neck extension after impact against the seat back)	2.65 (0.30)	100 (0)

**FIGURE 4:**
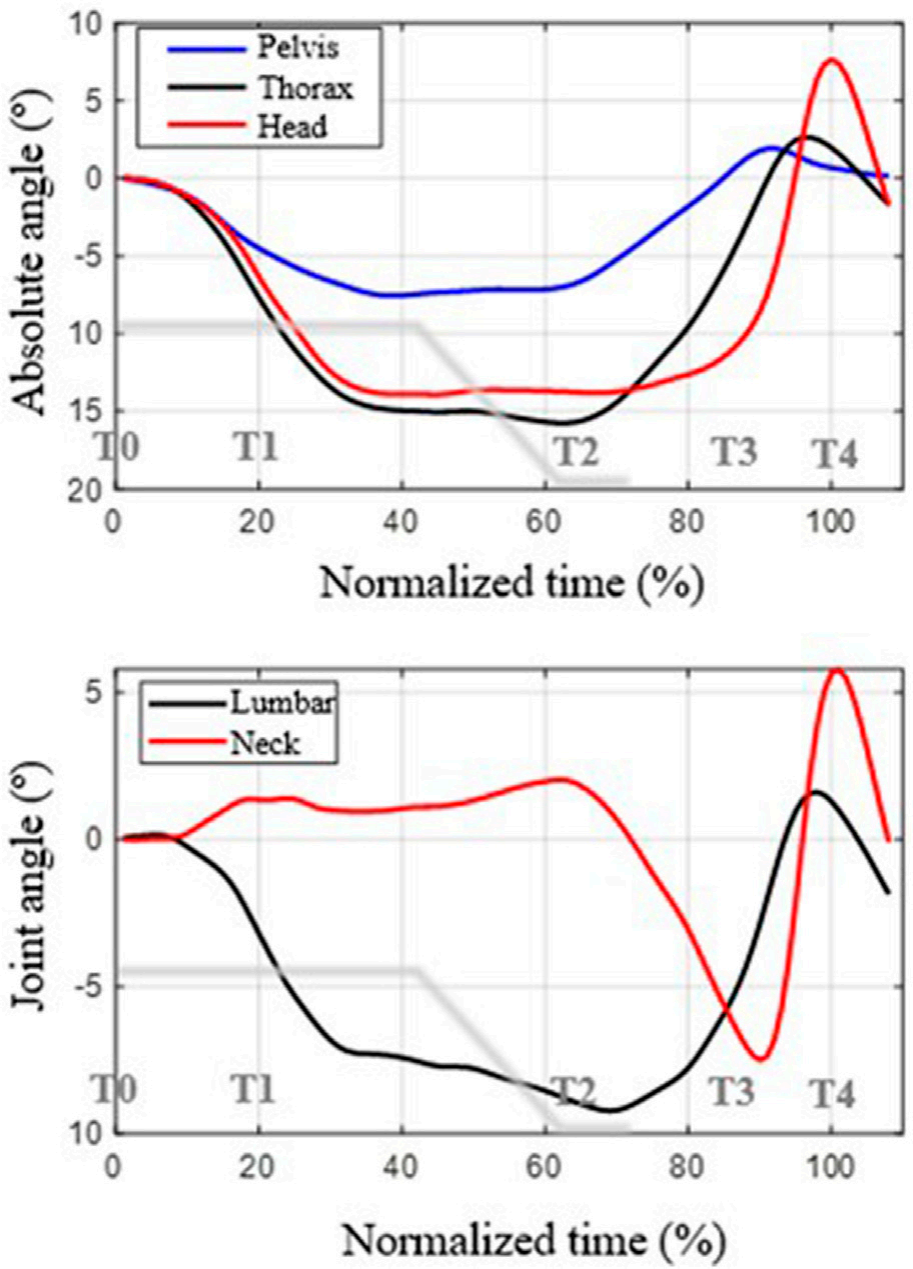
Description of the mean curves of the angles as a function of normalized time (the 100% instant corresponds to the maximum extension of the head). Top: rotation angles of each segment with respect to the reference position. Bottom: joint angles with respect to the reference position. Positive angles are backward rotation (counterclockwise, extension movement); negative angles are forward rotation (flexion movement). The deceleration curve of the vehicle during braking is shown in gray.

**FIGURE 5:**
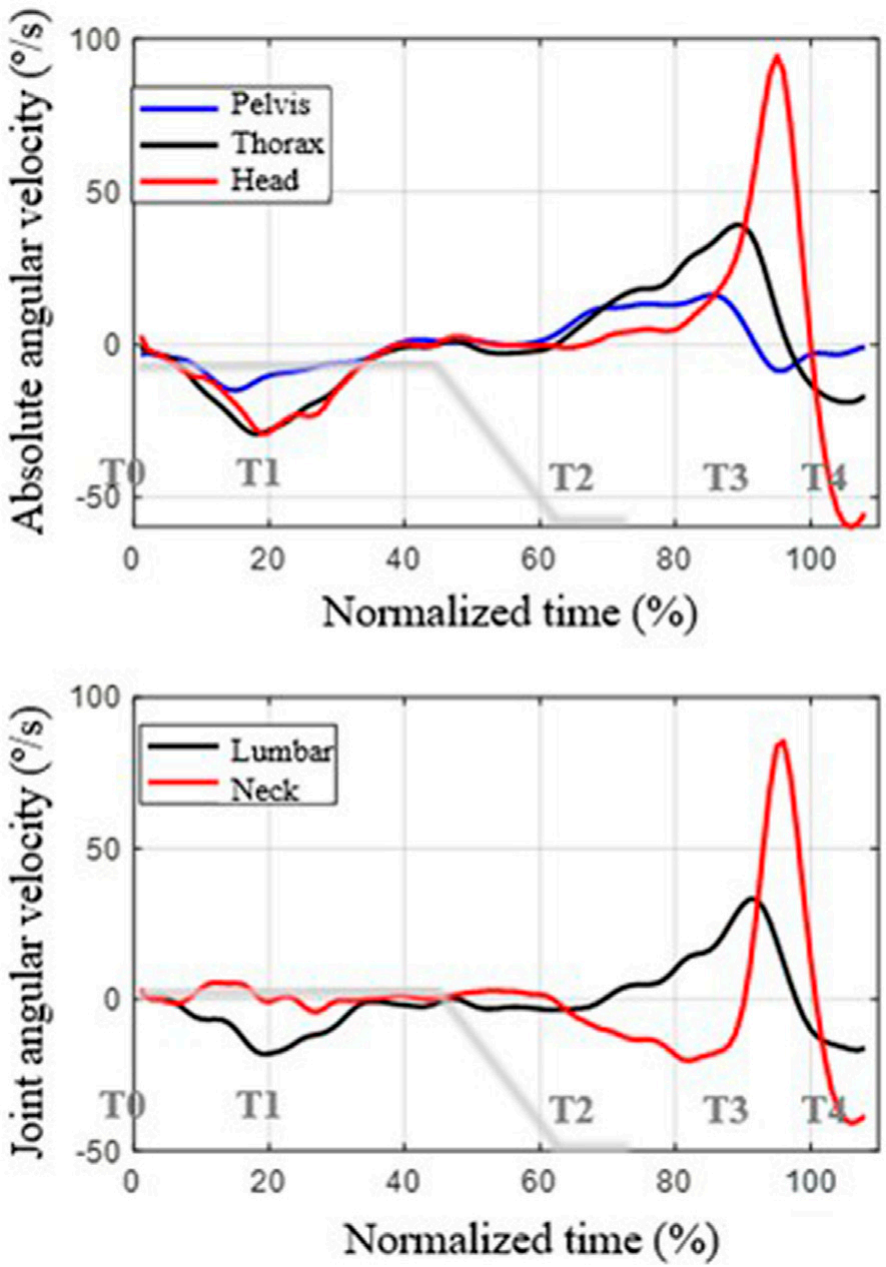
Mean functions of angular velocities across subjects as a function of normalized time (the instant t_n_ = 100 corresponds to the maximum extension of the head). Top: angular velocity of each segment. Bottom: angular velocity of the joint. Positive values represent counterclockwise angular velocity (extension movement); negative values represent forward rotation (flexion movement). The deceleration curve of the vehicle during braking is shown in gray.

The coordination between the movements of the three body segments (pelvis, thorax and head) and the joint angles are shown in Figures 4, 5, presenting the mean functions of the angles and angular velocities, respectively. Figure 4 shows the mean functions across subjects of the angles for each body segment. At the top are the absolute angles relative to the initial position at the beginning of braking. The lower part shows the angle of the joint, taking the initial position as a reference. Following the ISB (International Society of Biomechanics) sign convention, positive angles correspond to extension movement, while negative angles correspond to flexion movement (Wu et al., 2002). We will reference the thorax movement curve in the graph below (black curve) to describe the movement. As can be seen, at the beginning of braking (normalized instant t_n_ = 0%), a forward rotation occurs (flexion, negative thoracic angle), which reaches its maximum at around T2 (t_n_ = 62.9%). This instant coincides with the moment in which the vehicle stops. From this instant, the trunk rotates backward, recovering the initial angle, until it hits the back of the seat, slowing the movement of the thorax and inverting it at T3 (t_n_ = 86.5%).

As for the movement of the pelvis, its range is much smaller, and it is delayed compared to that of the thorax during braking. That is, the thorax drags the rotation of the pelvis. Since the pelvis rotates less than the thorax, some lumbar flexion appears, the range of which is around 10° on average.

Regarding the movement of the head, at the beginning of braking, there is a lag between its rotation and that of the thorax, which is slightly greater. This causes the neck not to flex but to extend slightly during the thoracic flexion movement. Neck flexion begins once the thorax has already started to move backward, after the instant t_n_ = 70%, due to the advancement of the thorax movement relative to that of the head. In the end, a very abrupt extension-flexion movement of the neck occurs due to the impact and posterior rebound of the trunk, from T3 (t_n_ = 86.5%), against the back of the seat and also due to the change in the center of head rotation.

Figure 5 shows the mean functions of angular velocities. The upper part of the figure shows the mean functions of the angular velocities of the pelvis, thorax and neck, while the lower part shows the mean velocities of the lumbar and neck joints. The gray line represents the braking deceleration curve.

The three segments move with negative angular velocity from the initial instant until approximately t_n_ = 20% (T1). Since the angular velocity of the thorax is greater than that of the pelvis, the lumbar angular velocity is not zero but flexion (a negative value). Between t_n_ = 20% (T1) and t_n_ = 40%, the angular velocities of the trunk and pelvis become almost zero until t_n_ = 60% (approx. T2), where the movements become an extension. The angular velocity of extension is more significant in the case of the thorax than in the pelvis from t_n_ = 70, so the lumbar angular velocity is positive from that instant until T3 (approx. t_n_ = 90%). Then, the impact of the thorax against the seat back occurs, which reverses the angular velocity of the two segments.

In the case of the head, between t_n_ = 0% (T0) and t_n_ = 20% (T1) its angular velocity is similar to that of the trunk, but it is slightly out of phase, so the motion of the neck results in extension. From that instant until t_n_ = 60% (approx. T2), both thorax and head move with small angular velocities, so the relative movement of the neck is practically null.

The return movement of the thorax corresponds to the normalized times between T2 and T3 (between t_n_ = 60% and t_n_ = 90%), approximately. In this interval, the angular velocity of the thorax is greater than that of the head, which means that the neck experiences flexion (negative velocity in the graph above).

When the thorax impacts against the seat back, it slows down, but the head undergoes an abrupt change in angular velocity, first with a peak of extension velocity and then with a peak of flexion. This abrupt change is linked to the change in the center of rotation of the neck, associated with the blocking of the movement of the thorax and pelvis while the neck continues to move.

Table 4 shows the parameters that numerically describe the curves in the above graphs. Note that the maximum and minimum values of the mean functions are lower than the averages of the maximum and minimum values of the individual curves due to a smoothing effect from the offset between curves. As can be seen, the movement with the most extensive range is that of the head. However, there is a notable angular displacement of the thorax and pelvis. The fastest movement is also that of the head, due to the abrupt movement after T3, the instant of impact of the back against the seat back.

**TABLE 4:** Numerical kinematic variables for the three segments. Mean (std). N = 17. Angular displacements and velocities are positive for extension movement and negative for flexion movement.

Variable	Pelvis	Thorax	Head
**Maximum flexion** (°)	−9.5 (6.7)	−19.1 (11.5)	−20.7 (12.6)
**Maximum extension** (°)	2.8 (2.2)	4.1 (2.8)	8.1 (5.2)
**Range of Flexion-Extension** (°)	12.3 (6.7)	23.2 (12.3)	28.8 (14.2)
**Maximum velocity flexion** (°/s)	−29.8 (24.1)	−44.3 (22.7)	−85.7 (45.3)
**Maximum velocity extension** (°/s)	33.7 (17.7)	61.1 (37.3)	111.8 (74.5)
**Range of velocity Flexion-Extension** (°/s)	63.5 (36.8)	105.4 (53.1)	197.5 (99.4)

To analyze the coordination between movements, the correlation coefficient between the ranges of movement of the pelvis on the seat, the lumbar joint (movement of the thorax relative to the pelvis) and the neck joint (movement of the head relative to the thorax) was calculated. The results are shown in Table 5.

**TABLE 5:** Spearman’s correlation coefficients between the mobility of each segment. Upper box, between angular ranges. Lower box (gray), between angular velocity ranges. Rho (*p*-value).

Body segment	Lumbar area	Neck
**Pelvis**	0.513 (0.035)	0.712 (0.001)
	0.659 (0.004)	0.860 (0.000)
**Lumbar area**		0.495 (0.043)
		0.807 (0.015)

It should be noted that neck range of motion is more closely related to pelvic rotation range than to lumbar flexion and extension range (rho of 0.712 *versus* 0.495). The correlation in the case of the angular velocity ranges (pelvis *versus* neck) is even greater, although only slightly higher than the lumbar-neck correlation (rho of 0.860 *versus* 0.807). These results show the potential effect of free movement of the pelvis on the range of motion of the neck and its angular velocity.

**FIGURE 6:**
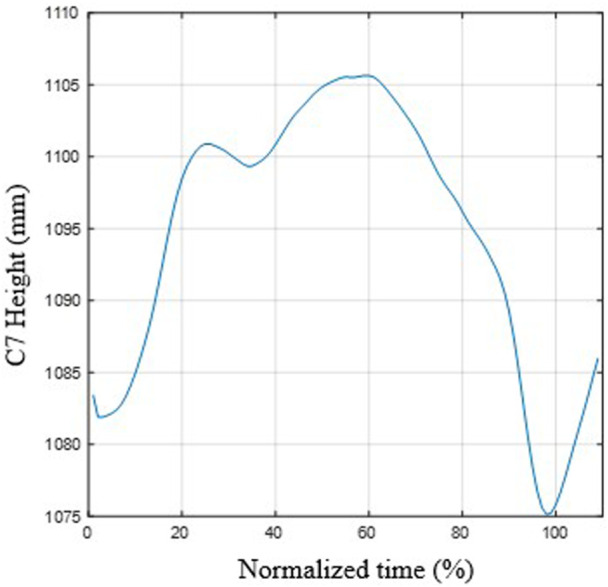
Mean function of the height of C7 during movement. The C7 marker increases during thoracic (and pelvic) flexion and decreases during the extension movement.

Figure 6 depicts the mean function of C7 marker height *versus* normalized time. As can be seen, the trunk rises during the flexion movement during braking (t_n_ from 0 to approximately 60%) and descends during the return movement. The mean elevation for the 17 subjects is 4.9 cm (std = 2.6 cm).

The rising of C7 is associated with both pelvic rotation and lumbar flexion, although these rotations do not contribute similarly. Indeed, Table 6 summarizes the results of the regression model of C7 height as a function of pelvic and lumbar flexion angles. The goodness of fit is good (median multiple correlation coefficient = 0.971), and the coefficient of the pelvic angle is four times greater than that of lumbar flexion (3.56 *versus* 0.88), with the difference being significant (*p* < 0.008 in Friedman’s test). These results demonstrate that the contribution of pelvic rotation to trunk elevation is more significant than that of lumbar flexion. This may be due to the elevation of the sacrum, S1, by rotation of the pelvis around an axis located at the ischial tuberosities (Page et al., 2009b). In the sitting position, the pelvis is tilted backward, and when turning forward, there is an elevation of the sacrum and, consequently, the entire trunk. However, this interpretation should be contrasted with more detailed kinematic studies.

**TABLE 6:** Coefficients of the regression models for height of C7 (t_i_) = a + b*pelvic rotation (t_i_) + c*lumbar rotation (t_i_), t_i_∈ [T_0_, T_2_]. N = 17. R = correlation coefficient; IQR = Interquartile range.

Coefficient	Median (IQR)
**b** (mm/°)	3.56 (4.03)
**c** (mm/°)	0.88 (3.25)
**R**	0.971 (0.13)

**FIGURE 7:**
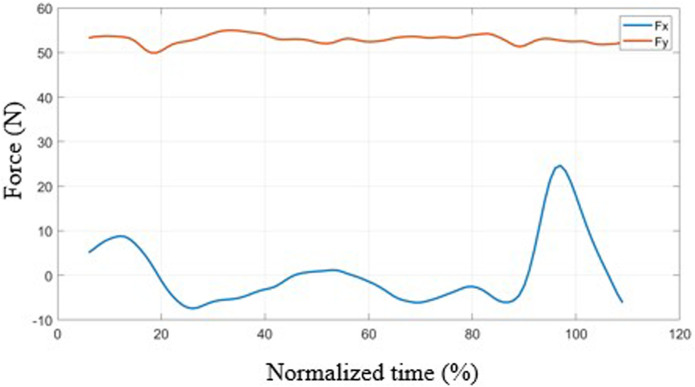
Mean force curves during movement as a function of normalized time.

Figure 7 shows the mean functions of the vertical and horizontal components of the net force that the neck exerts on the head. The vertical component takes an approximately constant value and is associated with the head’s weight, with small oscillations due to the vertical accelerations. In contrast, the horizontal forces peak at the end of the movement when the thorax impacts against the seat back, which stops the movement of the thorax and brakes the head. It should be noted that this peak occurs when the vehicle is entirely stationary so that the measured acceleration relative to the bus corresponds to the absolute acceleration. There is also a peak at the beginning of braking, which is much smaller. However, this occurs with the vehicle at negative acceleration so the absolute acceleration and force would be lower.

The average of the maximum forces in the horizontal direction is 30 N (std = 15.9 N), and in the vertical direction it is 61.5 N (std 10.6 N). The maximum value of the horizontal force in the mean function is lower than the average of the maxima due to the cancellation effect that appears due to the lag between curves (the curves have been aligned with the angles, not with the accelerations). The maximum horizontal forces range from 11.0 N to 57.6 N.

Finally, the risk of cervical injury was analyzed using the Neck Injury Criterion (NIC). The maximum value of the NIC does not appear during braking but in the impact of the thorax against the seat back during the return movement. The average of these maximum values was 0.69 m^2^/s^2^ (std = 0.40 m^2^/s^2^), with a range between 0.25 and 1.63 m^2^/s^2^. The tolerance level for assuming a neck injury is 15 m^2^/s2. The maximum NIC value obtained (1.63 m^2^/s^2^) is approximately nine times lower than the injury threshold, proving that the tests were designed at a safe level. These values may be higher with heavier braking.

## 4 Discussion

The study of unrestrained passengers’ body kinematics during braking has received limited attention in the literature, despite being a common scenario in public transportation and a potential scenario in autonomous vehicles, particularly at low speeds. While prior studies have predominantly focused on head-neck system dynamics, the comprehensive analysis of the entire kinematic chain, including pelvis-thorax-head movements, has been lacking. This gap may stem from the emphasis on restrained subjects in previous research, contrasting with the larger movements observed in unrestrained passengers during braking. When occupants are unrestrained and are subjected to braking (caused by a driver or an autonomous driving system), they experienced significant movements of both the neck and head, but also of the trunk if it is unrestrained, which translates into a movement of the pelvis that must also be considered.

While studies on trunk kinematics in seated positions exist, they primarily pertain to working postures characterized by voluntary and very slow movements, so angular velocities are low and accelerations are negligible (Vd et al., 2001; Page et al., 2009b). Although these studies highlight the role of the pelvis in the movement of the whole trunk, their results cannot be extrapolated to braking scenarios, where movements are much larger and faster and where coordination patterns are completely different. To our knowledge, this study represents the first experimental study to record and describe the movement of all three segments in unrestrained passengers (volunteers) during braking, thus precluding direct comparisons with previous findings. Previous research on body motion during traffic accidents (Panjabi, 1998; Ejima et al., 2007; Ejima et al., 2008; Behr et al., 2010; Carlsson and Davidsson, 2011; Ejima et al., 2012; Rooij et al., 2013; Ólafsdóttir et al., 2013; Östh et al., 2013; Kirschbichler et al., 2014; Kuo et al., 2018; Reed et al., 2018; Holt et al., 2020; Ghaffari and Davidsson, 2021; Larsson et al., 2022) has primarily focused on analyzing maximum head and torso excursions through linear positions and accelerations. In contrast, our study offers insights utilizing angular variables. Additionally, these prior studies have overlooked pelvic motion, rendering direct numerical comparisons unfeasible. Additionally, the authors posit that comparing maximum or average excursions could be misleading due to variations in experimental conditions and deceleration values across studies. It's worth noting that some of these studies have compared passengers restrained solely by a lap belt webbing against those secured by the standard three-point belt. Consistently, they (Bostrom and Haland, 2005; Siegmund et al., 2005; Parenteau, 2006; Carlsson and Davidsson, 2011; Khim et al., 2013; Ólafsdóttir et al., 2013; Östh et al., 2013; Kirschbichler et al., 2014; Larsson et al., 2022) have found that the chosen restraint system significantly influences the occupant’s kinematic response. The marked impact of the seat-and-belt combination on braking maneuvers can be attributed to the occupant’s substantial interaction with the upper portion of the three-point belt during braking maneuvers. When only a lap belt is utilized, a greater forward excursion of the trunk and head is observed, as neither of these areas is restricted. Moreover, in scenarios where passengers remain unrestrained, the pelvis plays a crucial role, given its lack of restraint. Past investigations on braking maneuvers have typically delineated two primary phases: a forward movement phase and a rebound phase. However, when subjects are restrained by a seat belt, the rebound phase tends to be less abrupt compared to observations in our study. This attenuation can be attributed to the support provided to the trunk and pelvis in restrained passengers, which limits significant forward movement, consequently reducing the potential for extensive backward motion during the rebound phase.

Our study reveals a consistent pattern amid individual variability, which can be interpreted as follows. During braking, the thorax, being the segment with the greatest mass, is propelled forward by the force of inertia, consequently dragging the pelvis and head with a slight delay relative to the thorax. This causes a slight neck extension and notable lumbar flexion. Subsequently, as the vehicle halts, the trunk undergoes a rearward movement, attributed to muscle compensation for the previous flexion. It is possible that the pitching effect of the vehicle also plays some role in this movement. However, both hypotheses will have to be tested in further studies.

One of the effects of pelvic rotation is that the upper part of the sacrum (S1) elevates, pushing and lifting the rest of the spine. This may be due to the pelvis being tilted backward in the starting position, as is usually the case in a relaxed sitting posture. Because the axis of rotation of the pelvis is approximately at the support of the ischial tuberosities (Page et al., 2009b), i.e., slightly above the seat and forward of the sacrum, the rotation of the pelvis is inevitably associated with an elevation of S1 and, with it, the rest of the trunk. This effect has been observed experimentally, with an elevation of C7 of several centimeters. This elevation is directly associated with pelvic rotation and not with trunk flexion, as demonstrated by the correlation analysis performed. This elevation, together with the slight extension of the neck in this phase, may cause compression of the neck, which should be analyzed in further studies.

Another pattern observed is the effect of the impact of the trunk against the seatback during the return movement post-braking after the vehicle has stopped. This impact abruptly halts thoracic and pelvic movements, causing a whiplash effect in the neck, evidenced by sudden angular velocity changes, with a consequent dynamic effect. This phenomenon has also been verified experimentally in angular velocity curves. It originates in impulsive actions associated with a sudden change in the position of the instantaneous center of rotation of the head. In fact, the head moves with a small angular velocity during return movement. This angular velocity is the sum of the angular velocity of the pelvis (positive), lumbar area (positive) and neck (negative) (Figure 5). Therefore, the position of the instantaneous center of head rotation must be below the lumbar area. Upon collision with the seat back, the lumbar and pelvic movements are blocked so that the displacement of the head is now fundamentally due to the movement of the neck, with a much smaller radius of gyration, which abruptly increases the angular velocity, producing a peak in the horizontal force (Figure 7).

The study conducted is a preliminary study with some important limitations. The first relates to the limited sample, although it was sufficient to be able to test the main hypotheses of the study. In addition, we have encountered problems with precision, due to camera vibrations, which have introduced some noise. This noise may affect the precision in the calculation of accelerations and position of instantaneous center of rotation of the pelvis and thorax. Consequently, dynamic analyses were not feasible in this study but should be addressed in future research endeavors.

In conclusion, our findings underscore the need to consider the kinematics and dynamics of the entire trunk when designing safety systems for vehicles and not only the head and neck, particularly in scenarios involving braking, where coordinated movement of all segments is evident. Furthermore, the non-negligible influence of pelvic movement on the overall kinematic chain emphasizes the importance of comprehensive analyses in enhancing passenger safety.

## 5 Conclusion

The analysis of unbelted passengers’ kinematics during low-speed braking in an autonomous bus reveals coordinated movement patterns involving thoracic flexion and extension, which drags the pelvis and head in its back-and-forth movements. Pelvic forward rotation results in the elevation of S1 and the entire spine. The contribution to C7 rise associated with pelvic rotation is much greater than the effect of lumbar flexion-extension.

The collision of the back against the seat back during the return movement induces a whiplash effect in the neck due to the abrupt change in the head’s center of rotation. This leads to sudden changes in its angular velocity, with dynamic effects that need to be studied in more detail. This study demonstrates that the rebounding motion and subsequent impacts against the seat could imply a higher risk situation that requires further study.

In conclusion, the study emphasizes that the design of safety systems in vehicles accommodating unbelted passengers should not solely focus on neck forces but also consider the coordinated movement of the entire kinematic chain, encompassing the torso and pelvis.

## Data Availability

The datasets presented in this article are not readily available because they include personal data. Requests to access the datasets should be directed to SS-C ssantos@ing.uc3m.es.

## References

[B1] Albert D. L. Beeman S. M. Kemper A. R. (2018). Occupant kinematics of the Hybrid III, THOR-M, and postmortem human surrogates under various restraint conditions in full-scale frontal sled tests. Traffic Inj. Prev. 19, S50–S58. 10.1080/15389588.2017.1405390 29584475

[B2] Albertsson P. Falkmer T. (2005). Is there a pattern in European bus and coach incidents? A literature analysis with special focus on injury causation and injury mechanisms. Accid. Anal. Prev. 37 (2), 225–233. 10.1016/j.aap.2004.03.006 15667808

[B3] Bansode R. S. Tanriover R. (2018). Global status report on road safety 2018; World health organization. Vol. 2, global status report on road safety 2018. Geneva: World Health Organization. Licence: CC BYNC-SA 3.0 IGO. 2018.

[B4] Barabino B. Eboli L. Mazzulla G. Mozzoni S. Murru R. Pungillo G. (2019). “An innovative methodology to define the bus comfort level,” in Transportation research procedia.

[B5] Beeman S. M. Kemper A. R. Madigan M. L. Duma S. M. (2011). Effects of bracing on human kinematics in low-speed frontal sled tests. Ann. Biomed. Eng. 39 (12), 2998–3010. 10.1007/s10439-011-0379-1 21870249

[B6] Beeman S. M. Kemper A. R. Madigan M. L. Franck C. T. Loftus S. C. (2012). Occupant kinematics in low-speed frontal sled tests: human volunteers, Hybrid III ATD, and PMHS. Accid. Anal. Prev. 47, 128–139. 10.1016/j.aap.2012.01.016 22342960

[B7] Behr M. Poumarat G. Serre T. Arnoux P. J. Thollon L. Brunet C. (2010). Posture and muscular behaviour in emergency braking: an experimental approach. Accid. Anal. Prev. 42 (3), 797–801. 10.1016/j.aap.2009.04.010 20380905

[B8] Björnstig U. Bylund P. O. Albertsson P. Falkmer T. Björnstig J. Petzäll J. (2005). INJURY events among BUS and coach occupants: non-crash injuries as important as crash injuries. IATSS Res. 29 (1), 79–87. 10.1016/s0386-1112(14)60121-7

[B9] Bose D. Crandall J. R. Untaroiu C. D. Maslen E. H. (2010). Influence of pre-collision occupant parameters on injury outcome in a frontal collision. Accid. Anal. Prev. 42 (4), 1398–1407. 10.1016/j.aap.2010.03.004 20441858

[B10] Bostrom O. Haland Y. (2005). Benefits of a 3+2-point belt system and an inboard torso side support in frontal, far-side and rollover crashes. Int. J. Veh. Saf. 1 (1–3), 181. 10.1504/ijvs.2005.007546

[B11] Carlsson S. Davidsson J., (2011). Volunteer occupant kinematics during driver initiated and autonomous braking when driving in real traffic environments, in: 2011 IRCOBI Conference Proceedings - International Research Council on the Biomechanics of Injury, Krakow, Poland, September 14-16, 2011.

[B12] De Graaf B. Van Weperen W. (1997). The retention of blance: an exploratory study into the limits of acceleration the human body can withstand without losing equilibrium. Hum. Factors 39 (1), 111–118. 10.1518/001872097778940614 9302883

[B13] Del Toro S. F. Santos-Cuadros S. Olmeda E. Román J. L. S. (2020). Study of the emergency braking test with an autonomous bus and the semg neck response by means of a low-cost system. Micromachines (Basel). 11 (10), 931. 10.3390/mi11100931 33066252 PMC7602115

[B14] Duhamel A. Bourriez J. L. Devos P. Krystkowiak P. Destée A. Derambure P. (2004). Statistical tools for clinical gait analysis. Gait Posture 20 (2), 204–212. 10.1016/j.gaitpost.2003.09.010 15336292

[B15] Eboli L. Mazzulla G. Pungillo G. (2016). “Measuring bus comfort levels by using acceleration instantaneous values,” in Transportation research procedia.

[B16] Ejima S. Ito D. Satou F. Mikami K. Ono K. Kaneoka K. (2012). “Effects of pre-impact swerving/steering on physical motion of the volunteer in the low-speed side-impact sled test,” in 2012 IRCOBI Conference Proceedings - International Research Council on the Biomechanics of Injury, Dublin (Ireland), 12 - 14 September 2012.

[B17] Ejima S. Ono K. Holcombe S. Kaneoka K. Fukushima M. (2007). “A study on occupant kinematics behaviour and muscle activities during pre-impact braking based on volunteer tests,” in International Research Council on the Biomechanics of Injury - 2007 International IRCOBI Conference on the Biomechanics of Injury, Proceedings, Porto, Portugal, 14 – 16 September 2022.

[B18] Ejima S. Zama Y. Satou F. Holcombe S. Ono K. Kaneoka K. (2008). “Prediction of the physical motion of the human body based on muscle activity during pre-impact braking,” in International Research Council on the Biomechanics of Injury - 2008 International IRCOBI Conference on the Biomechanics of Injury, Proceedings, Gothenburg (Sweden), 11 - 13 September 2013.

[B19] Ghaffari G. Davidsson J. (2021). Female kinematics and muscle responses in lane change and lane change with braking maneuvers. Traffic Inj. Prev. 22 (3), 236–241. 10.1080/15389588.2021.1881068 33688754

[B20] Graci V. Douglas E. Seacrist T. Kerrigan J. Mansfield J. Bolte J. (2019). Effect of automated versus manual emergency braking on rear seat adult and pediatric occupant precrash motion. Traffic Inj. Prev. 20, S106–S111. sup1. 10.1080/15389588.2019.1630734 31381438

[B21] Henezi D. Winkler A. (2023). “The role of public transport in transport safety and public safety,” in The eurasia proceedings of science Technology engineering and mathematics, 505–512. 23:Available from: http://www.epstem.net/en/pub/epstem/issue/79793/1374907 (Accessed January 8, 2024).

[B22] Hernández I. A. Fyfe K. R. Heo G. Major P. W. (2005). Kinematics of head movement in simulated low velocity rear-end impacts. Clin. Biomech. 20 (10), 1011–1018. 10.1016/j.clinbiomech.2005.07.002 16168533

[B23] Holt C. Seacrist T. Douglas E. Graci V. Kerrigan J. Kent R. (2020). The effect of vehicle countermeasures and age on human volunteer kinematics during evasive swerving events. Traffic Inj. Prev. 21 (1), 48–54. 10.1080/15389588.2019.1679798 31750733

[B24] Ito D. Ejima S. Kitajima S. Katoh R. Ito H. Sakane M. (2013). “Occupant kinematic behavior and effects of a motorized seatbelt on occupant restraint of human volunteers during low speed frontal impact: mini-sled tests with mass production car seat,” in 2013 IRCOBI Conference Proceedings - International Research Council on the Biomechanics of Injury, Gothenburg (Sweden), 11 - 13 September 2013.

[B25] Jakobsson L. Bergman T. Li J. (2006). “Identifying thoracic and lumbar spinal injuries in car accidents,” in International Research Council on the Biomechanics of Impact - 2006 International IRCOBI Conference on the Biomechanics of Impact, Proceedings, Madrid, Spain, September 2006.

[B26] Keller A. Krašna S. (2023). Accelerations of public transport vehicles: a method to derive representative generic pulses for passenger safety testing. Front. Future Transp. 4. 10.3389/ffutr.2023.931780

[B27] Khim J. Son C. Kim J. Jeon H. Ha J. Seo K. (2013). “A study of the relationship between seatbelt system and occupant injury in rear seat based on EuroNCAP frontal impact,” in Proceedings of the 23rd Enhanced Safety of Vehicles Conference, Seoul, South Korea, May 27-30, 2013.

[B28] Kirchner M. Schubert P. Haas C. T. (2014). Characterisation of real-world bus acceleration and deceleration signals. J. Signal Inf. Process. 05 (01), 8–13. 10.4236/jsip.2014.51002

[B29] Kirschbichler S. Huber P. Prüggler A. Steidl T. Sinz W. Mayer C. (2014). “Factors influencing occupant kinematics during braking and lane change maneuvers in a passenger vehicle,” in 2014 IRCOBI Conference Proceedings - International Research Council on the Biomechanics of Injury, Malaga (Spain), 14 – 16 September 2016.

[B30] Krašna S. Keller A. Linder A. Silvano A. P. Xu J. C. Thomson R. (2021). Human response to longitudinal perturbations of standing passengers on public transport during regular operation. Front. Bioeng. Biotechnol. 9, 680883. 10.3389/fbioe.2021.680883 34368094 PMC8343014

[B31] Kullgren A. Krafft M. (2007). “The effect of whiplash protection systems in real-life crashes and their correlation to consumer crash test programmes,” in Proc 20th ESV Conf(07-0468), Lyon (France), February 2007.

[B32] Kumar S. Ferrari R. Narayan Y. (2005a). Kinematic and electromyographic response to whiplash-type impacts. Effects of head rotation and trunk flexion: summary of research. Clin. Biomech. 20 (6), 553–568. 10.1016/j.clinbiomech.2005.02.007 15927733

[B33] Kumar S. Ferrari R. Narayan Y. (2005b). Kinematic and electromyographic response to whiplash loading in low-velocity whiplash impacts - a review. Clin. Biomech. 20 (4), 343–356. 10.1016/j.clinbiomech.2004.11.016 15737441

[B34] Kumar S. Ferrari R. Narayan Y. Jones T. (2006). The effect of seat belt use on the cervical electromyogram response to whiplash-type impacts. J. Manip. Physiol. Ther. 29 (2), 115–125. 10.1016/j.jmpt.2005.12.008 16461170

[B35] Kumar S. Narayan Y. Amell T. (2002). An electromyographic study of low-velocity rear-end impacts. Spine (Phila Pa 1976) 27 (10), 1044–1055. 10.1097/00007632-200205150-00009 12004171

[B36] Kumar S. Narayan Y. Amell T. (2003a). Analysis of low velocity frontal impacts. Bristol, Avon: Clin Biomech. Available from: http://www.ncbi.nlm.nih.gov/pubmed/12957555 (Accessed February 3, 2020). 10.1016/s0268-0033(03)00137-2 12957555

[B37] Kumar S. Narayan Y. Amell T. (2003b). Analysis of low velocity frontal impacts. Clin. Biomech. 18 (8), 694–703. 10.1016/s0268-0033(03)00137-2 12957555

[B38] Kuo C. Fanton M. Wu L. Camarillo D. (2018). Spinal constraint modulates head instantaneous center of rotation and dictates head angular motion. J. Biomech. 76, 220–228. 10.1016/j.jbiomech.2018.05.024 29929891

[B39] Larsson E. Ghaffari G. Iraeus J. Davidsson J. (2022). “Passenger kinematics variance in different vehicle manoeuvres - biomechanical response corridors based on principal component analysis,” in Conference proceedings International Research Council on the Biomechanics of Injury, IRCOBI, Porto, Portugal, 14 – 16 September 2022.

[B40] Lutin J. M. Kornhauser A. L. Spears J. Sanders L. F. (2016). “A research roadmap for substantially improving safety for transit buses through autonomous braking assistance for operators,” in Transportation Research Board 95th Annual Meeting, Washington DC, United States, January 10–14, 2016.

[B41] Malm S. Krafft M. Kullgren A. Ydenius A. Tingvall C. (2008). “Risk of permanent medical impairment (RPMI) in road traffic accidents,” in Annals of Advances in Automotive Medicine - 52nd Annual Scientific Conference, San Diego, California, October 2008. PMC3256772 19026226

[B42] McConnell W. E. Howard R. P. Guzman H. M. Bomar J. B. Raddin J. H. Benedict J. V. (1993). “Analysis of human test subject kinematic responses to low velocity rear end impacts,” in SAE technical papers (Pennsylvania, United States: SAE International).

[B43] McConnell W. E. Howard R. P. Poppel J. V. Krause R. Guzman H. M. Bomar J. B. (1995). “Human head and neck kinematics after low velocity rear-end impacts - understanding “whiplash.”,” SAE Int. J. Passeng. Cars 104 (6), 3106–3129. (SAE International).

[B44] McMurry T. L. Poplin G. S. Shaw G. Panzer M. B. (2018). Crash safety concerns for out-of-position occupant postures: a look toward safety in highly automated vehicles. Traffic Inj. Prev. 19 (6), 582–587. 10.1080/15389588.2018.1458306 29630403

[B45] Meyer F. Humm J. Purushothaman Y. Willinger R. Pintar F. A. Yoganandan N. (2019). Forces and moments in cervical spinal column segments in frontal impacts using finite element modeling and human cadaver tests. J. Mech. Behav. Biomed. Mater 90, 681–688. 10.1016/j.jmbbm.2018.09.043 30529569

[B46] Ólafsdóttir J. M. Östh J. K. H. Davidsson J. Brolin K. B. (2013). “Passenger kinematics and muscle responses in autonomous braking events with standard and reversible pre-tensioned restraints,” in 2013 IRCOBI Conference Proceedings - International Research Council on the Biomechanics of Injury, Gothenburg (Sweden), 11 - 13 September 2013.

[B47] Olivares G. (2015). Crashworthiness evaluation of mass transit buses. Accid. Reconstr. J. 25 (4). 10.21949/1503559

[B48] Olivares G. Yadav V. (2009). “Injury mechanisms to mass transit bus passengers during frontal, side and rear impact crash scenarios,” in PROCEEDINGS OF THE 21ST (ESV) INTERNATIONAL TECHNICAL CONFERENCE ON THE ENHANCED SAFETY OF VEHICLES, STUTTGART, GERMANY, JUNE 2009.

[B49] Olive O. D. Marianne M. Henry P. M. Hung-Kay C. D. (2019). Muscle activity during low-speed rear impact. Chin. J. Traumatology - Engl. Ed. 22 (2), 80–84. 10.1016/j.cjtee.2018.10.006 PMC6487461 30962127

[B50] Ono K. Kanno M. (1996). Influences of the physical parameters on the risk to neck injuries in low impact speed rear-end collisions. Accid. Anal. Prev. 28 (4), 493–499. 10.1016/0001-4575(96)00019-x 8870776

[B51] Östh J. Ólafsdóttir J. M. Davidsson J. Brolin K. (2013). Driver kinematic and muscle responses in braking events with standard and reversible pre-tensioned restraints: validation data for human models. Stapp Car Crash J. 57, 1–41. 10.4271/2013-22-0001 24435725

[B52] Page A. Candelas P. Belmar F. (2006). On the use of local fitting techniques for the analysis of physical dynamic systems. Eur. J. Phys. 27 (2), 273–279. 10.1088/0143-0807/27/2/010

[B53] Page Á. De Rosario H. Mata V. Atienza C. (2009a). Experimental analysis of rigid body motion. A vector method to determine finite and infinitesimal displacements from point coordinates. J. Mech. Des. 131 (3). 10.1115/1.3066468

[B54] Page A. de Rosario H. Mata V. Porcar R. Solaz J. Such M. J. (2009b). Kinematics of the trunk in sitting posture: an analysis based on the instantaneous axis of rotation. Ergonomics 52 (6), 695–706. 10.1080/00140130802559001 19479581

[B55] Palacio A. Tamburro G. O’Neill D. Simms C. K. (2009). Non-collision injuries in urban buses—strategies for prevention. Accid. Anal. Prev. 41 (1), 1–9. 10.1016/j.aap.2008.08.016 19114131

[B56] Panjabi M. M. (1998). Cervical spine models for biomechanical research. Spine (Phila Pa 1976) 23 (24), 2684–2699. 10.1097/00007632-199812150-00007 9879095

[B57] Panjabi M. M. Pearson A. M. Ito S. Ivancic P. C. Wang J. L. (2004). Cervical spine curvature during simulated whiplash. Clin. Biomech. 19 (1), 1–9. 10.1016/j.clinbiomech.2003.09.006 14659923

[B58] Parenteau C. (2006). A comparison of volunteers and dummy upper torso kinematics with and without shoulder belt slack in a low speed side/pre-roll environment. Traffic Inj. Prev. 7 (2), 155–163. 10.1080/15389580500481973 16854710

[B59] Pons J. L. Moreno J. C. Torricelli D. Taylor J. S. (2013). “Principles of human locomotion: a review,” in 2013 35th Annual International Conference of the IEEE Engineering in Medicine and Biology Society (EMBC), Osaka, Japan, 3-7 July 2013 (IEEE), 6941–6944. 10.1109/EMBC.2013.6611154 24111341

[B60] Ramsay J. O. (1997). SBW. Functional data analysis. New York: Springer.

[B61] Reed M. P. Ebert S. M. Jones M. L. H. Park B. K. D. Hallman J. J. Sherony R. (2018). Passenger head kinematics in abrupt braking and lane change events. Traffic Inj. Prev. 19, S70–S77. sup2. 10.1080/15389588.2018.1481957 30543309

[B62] Rooij L. van Elrofai H. Philippens M. M. Daanen H. A. (2013). Volunteer kinematics and reaction in lateral emergency maneuver tests. Stapp Car Crash J. 57, 313–342. 10.4271/2013-22-0013 24435737

[B63] Santos cuadros S. Fuentes Del Toro S. Olmeda E. San Román J. L. (2021). Surface electromyography study using a low‐cost system: are there neck muscles differences when the passenger is warned during an emergency braking inside an autonomous vehicle? Sensors 21 (16), 5378. 10.3390/s21165378 34450818 PMC8399791

[B64] Schubert P. Liebherr M. Kersten S. Haas C. T. (2017). Biomechanical demand analysis of older passengers in a standing position during bus transport. J. Transp. Health 4, 226–236. 10.1016/j.jth.2016.12.002

[B65] Siegmund G. P. Chimich D. D. Heinrichs B. E. Demarco A. L. Brault J. R. (2005). Variations in occupant response with seat belt slack and anchor location during moderate frontal impacts. Traffic Inj. Prev. 6 (1), 38–43. 10.1080/15389580590903159 15823873

[B66] Siegmund G. P. Sanderson D. J. Myers B. S. Inglis J. T. (2003). Rapid neck muscle adaptation alters the head kinematics of aware and unaware subjects undergoing multiple whiplash-like perturbations. J. Biomech. 36 (4), 473–482. 10.1016/s0021-9290(02)00458-x 12600337

[B67] Soule H. H. Huck S. Krum A. Wang Y. Ke R. Valadez D. (2020). Testing an automated collision avoidance and emergency braking system for buses. Transp. Res. Rec. 2674 (4), 66–74. 10.1177/0361198120912431

[B68] Svensson M. Y. Aldman B. Hansson H. A. Håland Y. Lövsund P. Boström O. (1996). A new neck injury criterion candidate-based on injury findings in the cervical spinal ganglia after experimental neck extension trauma, 123–136. Available from: http://publications.lib.chalmers.se/publication/181305-a-new-neck-injury-criterion-candidate-based-on-injury-findings-in-the-cervical-spinal-ganglia-after .

[B69] Szabo T. J. Welcher J. B. (1996). “Human subject kinematics and electromyographic activity during low speed rear impacts,” in SAE technical papers (Pennsylvania, United States: SAE International).

[B70] Toro W. R. O. (2022). Validación de un modelo dinámico del cuello para aplicaciones en Ergonomía y Valoración Funcional.

[B71] Union PO of the E (2018). Cost-effectiveness analysis of policy options for the mandatory implementation of different sets of vehicle safety measures: review of the General Safety and Pedestrian Safety Regulations: technical annex to GSR2 report SI2.733025: final report. Available from: https://op.europa.eu/en/publication-detail/-/publication/ed4aff17-49c5-11e8-be1d-01aa75ed71a1/language-en (Accessed January 9, 2024).

[B72] Vd J. H. Dl M. P. Hermans V. (2001). Effects of dynamic office chairs on trunk kinematics, trunk extensor EMG and spinal shrinkage. Ergonomics 44 (7), 739–750. 10.1080/00140130110045794 11437206

[B73] Venegas W. Inglés M. Page Á. Serra-Añó P. (2020). Paths of the cervical instantaneous axis of rotation during active movements—patterns and reliability. Med. Biol. Eng. Comput. 58 (5), 1147–1157. 10.1007/s11517-020-02153-5 32193862

[B74] Vychytil J. Špirk S. (2020). Numerical analysis of passenger kinematics and injury risks during a railway vehicle collision: the effect of safety belts. Appl. Comput. Mech. 14 (1). 10.24132/acm.2020.562

[B75] Watanabe Y. Ichikawa H. Kayama O. Ono K. Kaneoka K. Inami S. (2000). Influence of seat characteristics on occupant motion in low-speed rear impacts. Accid. Anal. Prev. 32 (2), 243–250. 10.1016/s0001-4575(99)00082-2 10688480

[B76] Wu G. Siegler S. Allard P. Kirtley C. Leardini A. Rosenbaum D. (2002). ISB recommendation on definitions of joint coordinate system of various joints for the reporting of human joint motion - Part I: ankle, hip, and spine. J. Biomechanics 35, 543–548. 10.1016/s0021-9290(01)00222-6 11934426

[B77] Yoganandan N. Pintar F. A. Banerjee A. (2017). Load-based lower neck injury criteria for females from rear impact from cadaver experiments. Ann. Biomed. Eng. 45 (5), 1194–1203. 10.1007/s10439-016-1773-5 28091968

