# Supplementary material for: Kinematic analysis of an unrestrained passenger in an autonomous vehicle during emergency braking
Source: Front Bioeng Biotechnol. 2024 Mar 12;12:1270181. doi: 10.3389/fbioe.2024.1270181 (PMC10963408; doi:10.3389/fbioe.2024.1270181)
Supplement: Supplementary file 1 [file DataSheet1.docx]

Supplementary Material

# Supplementary Figures and Tables

## Supplementary Figures

##
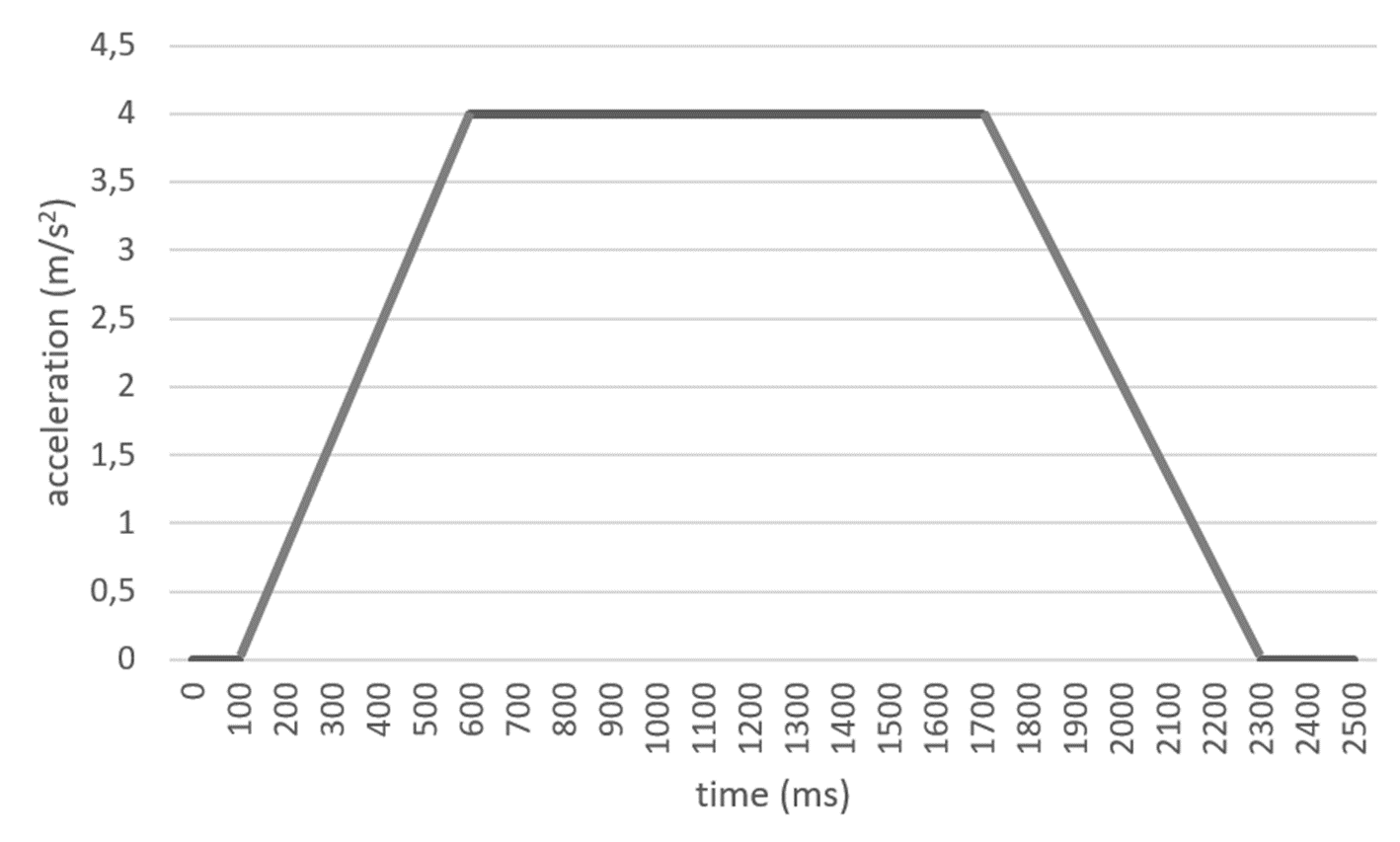


**Supplementary Figure 1.** Deceleration curve programmed in the autonomous vehicle during emergency braking.


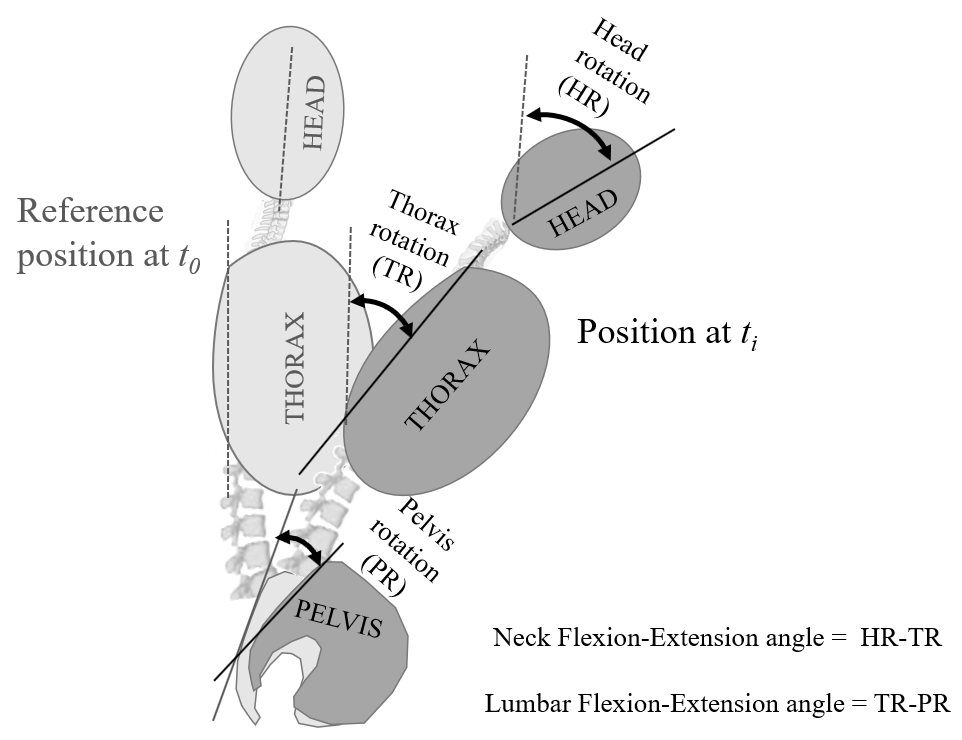


**Supplementary Figure 2.** Measured angles. The rotation angles of each segment (pelvis, thorax and head) are measured from the angular displacement of the technical markers from the reference position at time t0 to the position at time ti. Clockwise turns (flexion) are negative; counterclockwise turns (extension) are positive. The flexion-extension angle of the neck is obtained by subtracting the rotation of the thorax from that of the head. The lumbar flexion-extension angle is obtained by subtracting the rotation of the pelvis from that of the thorax. Angles relative to anatomical landmarks are not taken; the neck or lumbar joint angles are measured from the initial reference position.

##
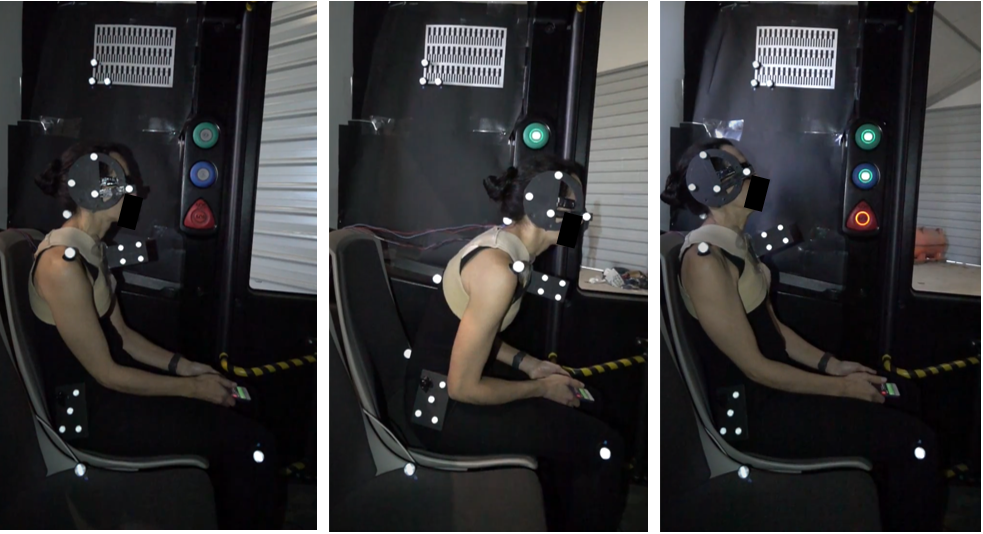


## Supplementary Figure 3. Movement of a subject during the emergency braking test (from left to right: starting position (T0), the instant of maximum thorax flexion (T2), the instant of maximum head extension (T4)).


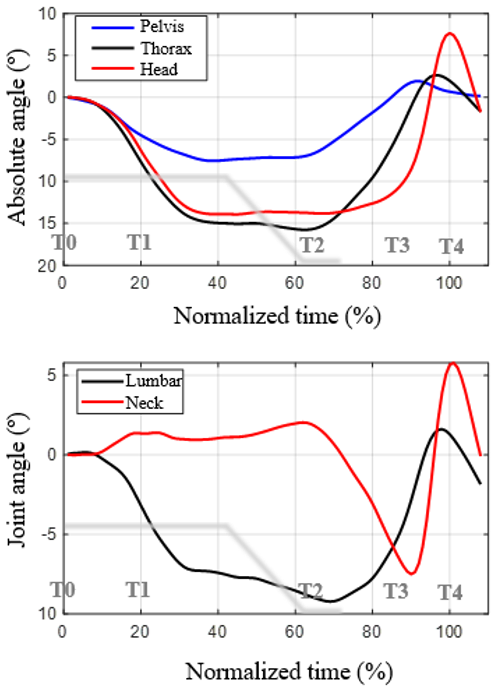


**Supplementary Figure 4.** Description of the mean curves of the angles as a function of normalized time (the 100% instant corresponds to the maximum extension of the head). Top: rotation angles of each segment with respect to the reference position. Bottom: joint angles with respect to the reference position. Positive angles are backward rotation (counterclockwise, extension movement); negative angles are forward rotation (flexion movement). The deceleration curve of the vehicle during braking is shown in gray.


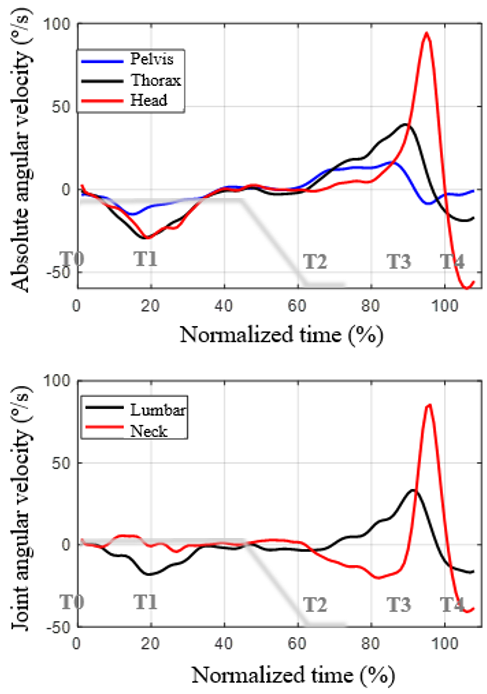


**Supplementary Figure 5.** Mean functions of angular velocities across subjects as a function of normalized time (the instant tn = 100 corresponds to the maximum extension of the head). Top: angular velocity of each segment. Bottom: angular velocity of the joint. Positive values represent counterclockwise angular velocity (extension movement); negative values represent forward rotation (flexion movement). The deceleration curve of the vehicle during braking is shown in gray.


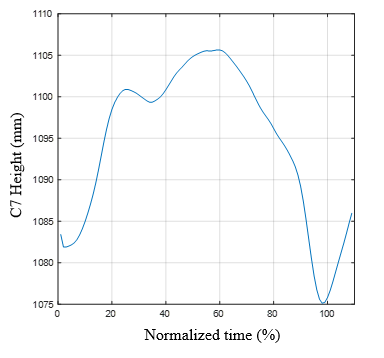


**Supplementary Figure 6.** Mean function of the height of C7 during movement. The C7 marker increases during thoracic (and pelvic) flexion and decreases during the extension movement.
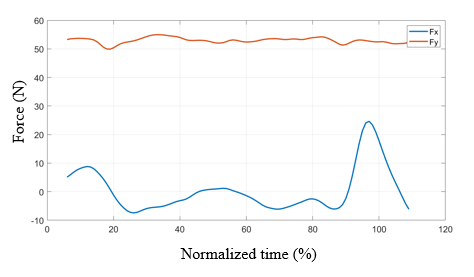


**Supplementary Figure 7.** Mean forces curves during movement as a function of normalised time.

## Supplementary Tables

**Table 1:** Kinematic, functional and numerical variables that appear in the analysis.

| **Magnitude** | **Functional variables** | **Numerical variables** |
| --- | --- | --- |
| **Angles** (°) | Head rotation  Thorax rotation  Pelvis rotation  Neck flexo-extension angle  Lumbar flexo-extension angle | Maximum head flexion and extension  Head flexo-extension range  Maximum thorax flexion and extension  Thorax flexo-extension range  Maximum pelvis flexion and extension  Pelvis rotation range |
| **Displacements** | Displacement of C7  Vertical displacement of the auricular marker with respect to C7 | Range |
| **Neck length** | Vertical distance between C7 and the auricular (ear) canal |  |
| **Angular velocities** (°/s) | Head angular velocity  Thorax angular velocity  Pelvis angular velocity  Neck flexo-extension angular velocity  Lumbar flexo-extension angular velocity | Maximum head flexion velocity  Maximum head extension velocity  Head flexo-extension velocity range  Maximum thorax flexion velocity  Maximum thorax extension velocity  Thorax flexo-extension velocity range  Maximum pelvis flexion velocity  Maximum pelvis extension velocity  Head flexo-extension velocity range |

**Table 2:** Characteristics of the participants in the study.

| **FEATURE** | **MALE (n=10)** | **FEMALE (n=7)** | **TOTAL (n=17)** |
| --- | --- | --- | --- |
| **Age** (years) | 29.2 (10.19) | 35.4 (6.9) | 31.7 (9.3) |
| **Height** (cm) | 177.5 (6.3) | 161.9 (7.9) | 171.1 (10.4) |
| **Weight** (kg) | 70.1 (12.2) | 61.2 (15.4) | 66.4 (13.8) |
| **Length neck C7-Tragus** (cm) | 10.8 (1.5) | 11.1 (1.8) | 10.9 (1.6) |
| **Length pelvis-C7** (mm) | 52.5 (1.5) | 46.6 (5.8) | 50.1 (4.8) |

**Table 3:** Time instants of the events taken as reference points.

| **Event** | **Time (s) from T_0_. Mean (std)** | **Normalized time t_n_ (%) from T_0._ Mean (std)** |
| --- | --- | --- |
| **T_1_** (Maximum angular velocity of thorax flexion) | 0.46 (0.23) | 20.4 (6.8) |
| **T_2_** (Maximum thorax flexion) | 1.66 (0.33) | 62.9 (11.8) |
| **T_3_** (Maximum angular velocity of the thorax before tn = 100) | 2.27 (0.21) | 86.5 (6.0) |
| **T_4_** (Maximum neck extension after impact against the seat back) | 2.65 (0.30) | 100 (0) |

**Table 4:** Numerical kinematic variables for the three segments. Mean (std). N = 17. Angular displacements and velocities are positive for extension movement and negative for flexion movement.

| **VARIABLE** | **Pelvis** | **Thorax** | **Head** |
| --- | --- | --- | --- |
| **Maximum flexion** (°) | −9.5 (6.7) | −19.1 (11.5) | −20.7 (12.6) |
| **Maximum extension (°)** | 2.8 (2.2) | 4.1 (2.8) | 8.1 (5.2) |
| **Range of Flexion-Extension** (°) | 12.3 (6.7) | 23.2 (12.3) | 28.8 (14.2) |
| **Maximum velocity flexion** (°/s) | −29.8 (24.1) | −44.3 (22.7) | −85.7 (45.3) |
| **Maximum velocity extension** (°/s) | 33.7 (17.7) | 61.1 (37.3) | 111.8 (74.5) |
| **Range of velocity Flexion-Extension** (°/s) | 63.5 (36.8) | 105.4 (53.1) | 197.5(99.4) |

**Table 5:** Spearman’s correlation coefficients between the mobility of each segment. Upper box, between angular ranges. Lower box (gray), between angular velocity ranges. Rho (p-value).

| **BODY SEGMENT** | **Lumbar area** | **Neck** |
| --- | --- | --- |
| **Pelvis** | 0.513 (0.035) | 0.712 (0.001) |
|  | 0.659 (0.004) | 0.860 (0.000) |
| **Lumbar area** |  | 0.495 (0.043) |
|  |  | 0.807 (0.015) |

**Table 6:** Coefficients of the regression models for height of C7(ti)= a + b*pelvic rotation(ti) + c*lumbar rotation(ti), ti [T0, T2]. N = 17. R = correlation coefficient; IQR = Interquartile range.

| **Coefficient** | **Median (IQR)** |
| --- | --- |
| **b** (mm/º) | 3.56 (4.03) |
| **c** (mm/º) | 0.88 (3.25) |
| **R** | 0.971 (0.13) |
